# Tensile Load-Bearing Behaviour of Concrete Components Reinforced with Flax Fibre Textiles

**DOI:** 10.3390/ma17061313

**Published:** 2024-03-12

**Authors:** Marcus Ricker, Sebastian Kuhn, Tânia Feiri, Katrin Zecherle, Jan Binde, Jana Winkelmann

**Affiliations:** 1 Chair of Structural Concrete, Department of Architecture and Civil Engineering, TU Dortmund University, 44227 Dortmund, Germany; sebastian.kuhn@tu-dortmund.de (S.K.); tania.feiri@tu-dortmund.de (T.F.); 2 Institute of Structural Engineering, Department of Civil Engineering and Project Management, Hochschule Biberach—University of Applied Sciences, 88400 Biberach an der Riß, Germany; 3 Department of Water, Environment, Construction and Safety—Hochschule Magdeburg-Stendal, 39114 Magdeburg, Germany; jan.binde@h2.de; 4 Fraunhofer Institute for Wood Research, 38108 Braunschweig, Germany; jana.winkelmann@wki.fraunhofer.de

**Keywords:** textile-reinforced concrete, flax fibre textiles, bio-based impregnation, leno fabrics, tensile tests

## Abstract

In recent years, the use of natural flax fibres as a reinforcement in composite building structures has witnessed a growing interest amongst research communities due to their green, economical, and capable mechanical properties. Most of the previous investigations on the load-bearing behaviour of concrete components reinforced with natural flax fibres include inorganic impregnations (or even no impregnation) and exclude the use of textile fabrics. Also, the mechanical behaviour of textiles made of natural flax fibres produced as leno fabrics remains to be investigated. In this paper, the results of tensile tests on concrete components reinforced with bio-based impregnated leno fabrics are presented. For comparison, multilayer non-impregnated and impregnated textiles were considered. The results demonstrated that reinforced textiles yielded an increase in the failure loads compared to the concrete cross-sections without reinforcement. The stress-strain diagrams showed that the curves can be divided into three sections, which are typical for reinforced tensile test specimens. For the impregnated textiles, a narrowly distributed crack pattern was observed. The results showed that impregnated textiles tend to support higher failure stresses with less strains than non-impregnated textiles. Moreover, an increase in the reinforcement ratio alongside larger opening widths of the warp yarns enables higher failure loads.

## 1. Introduction

In recent years, the growing ecological, social and economic awareness and the intent to save petroleum resources and reduce CO_2_ emissions have stimulated the search for green materials compatible with the environment [1]. In the construction sector, alternative and more ecological and sustainability-friendly materials are needed not only for new buildings and infrastructure, but also to extend the lifetime of the existing built environment.

Over the last decades, distinct reinforcement materials embedded in cement matrices have been investigated. Pioneering research has been conducted on the structural behaviour of non-metallic reinforcements (i.e., textile reinforcements) subjected to various types of loadings and incorporating different types of synthetic fibres (e.g., glass or carbon fibres) in distinct sizes and shapes (e.g., [2,3,4,5,6,7,8,9,10,11,12]). The results suggest that reinforcements with synthetic fibres are promising alternatives to conventional reinforcing steel due to their satisfactory physical and mechanical properties in terms of load bearing capacity and flexural performance. Also, the excellent corrosion resistance of the technical fabrics enables the reduction of the required concrete cover without affecting the load-bearing capacity and the durability of concrete components. This permits the reduction of the necessary reinforcement and concrete cross-section [13]. From an environmental perspective, reduced dimensions can generate significant cement savings—and, ultimately, lower CO_2_ emissions. These properties have been supporting the development of slimmer and more lightweight applications than possible with regular steel-reinforced concrete components (e.g., pedestrian bridges and facade panels) [2,3,4,5].

Other research developments have been contributing to the unlocking of the potential of non-metallic reinforcements and encouraging its adoption in concrete components. In Germany, for example, two research projects conducted by TUD Dresden University of Technology and RWTH Aachen University—SFB 528 and SFB 532—have been investigating the load-bearing behaviour of concrete elements reinforced with Alkali Resistant glass fibre textiles (AR-glass) or carbon fibre textiles (e.g., [6,7,8,9]). Also, in the context of the research project C^3^ (Carbon Concrete Composite), an innovative construction method for textile-reinforced concrete has been established [10] alongside optimised design provisions for new reinforcement types [11,12]. This project aims to implement a guideline for the design of textile-reinforced structural elements [14] with the goal to replace (at least parts of) the lengthy experimental investigations required for the verification of load-bearing capacity with practical design provisions [15,16,17]. Yet, the use of natural fibres—which are also known, at least since the 1980s [18], to be green, economical, and have promising mechanical properties for use as a reinforcement in cement-based matrices [19]—have been left outside the scope of these research developments in the domain of synthetic fibres.

Over the last few years, remarkable advances have been made to integrate natural fibres into existing concrete technologies. For example, Li et al. (e.g., [20,21,22]) have conducted extensive investigations in the field of engineered cementitious composites (ECC), a class of ultra-ductile/bendable fibre-reinforced cementitious composites developed in the 1990s (based on technology from the 1970s) for applications in the construction industry. These researchers have demonstrated that plant fibres (e.g., the renewable curauá plant fibre growing in Amazon, Brazil [23]) have the potential to replace synthetic fibres in the ECC composites due to their lightweight properties, low thermal conductivity, and material toughness derived from a combination of tensile strength and tensile ductility; thus, emerging as promising solutions for building façade and cladding applications.

Among the wide range of natural fibres, flax fibres—extracted from the bast or skin of the flax plants stem—seem to stand out due to their ecological properties such as their nearly neutral carbon account, their low-embodied energy use, their end-of-life harmless consequences for the environment even after degradation (e.g., [24,25]), as well as their harmless properties for human health [26]. Natural flax fibres are soft and flexible due to their high length–diameter ratio; they also have high cellulose content and some studies claim that they might have promising tensile strength properties for being adopted as a reinforcement embedded in cement-based matrices (e.g., [24,27,28]). These favourable characteristics have motivated the development of potential structural applications. For example, the Fraunhofer Institute for Wood Research (Wilhelm-Klauditz-Institut, WKI in Germany) presented at the BAU 2019 (a reputable trade fair for architecture, construction materials and systems in Germany) a prototype of a walkway built as a concrete slab reinforced with impregnated flax fibre textiles. With this prototype, a satisfactory fibre–concrete bond was shown to be possible, and it was demonstrated that natural flax fibres, as reinforcement, can result in promising resource-saving, cost-effective and durable solutions [25]. Other applications seem to have potential; for example, Trochoutsou et al. [27] demonstrated that flax fibres embedded in inorganic matrices can be utilised as a retrofitting solution for masonry structures.

However, as unlikely conventional synthetic fibres, flax fibres have significantly greater scatter in their mechanical properties (e.g., as in wood) due to the potential degradation arising from the production processes and measurement deviations in terms of moisture, temperature and others (e.g., plant growth, harvesting stage, fibre extraction stage, supply stage, measurement conditions and surface treatment) (e.g., [29,30,31]). Moreover, flax fibres are susceptible to volume changes caused by their hydrophilic nature, which can negatively impact their adhesion to the cementitious matrix—and, ultimately, the overall mechanical properties of the composite (e.g., [24,32,33,34]). In this context, some authors demonstrated that specific cement compositions (e.g., high amounts of Portland cement replacement with metakaolin [35]) may contribute to avoiding fibre degradation and embrittlement. Other studies, as Cevallos and Olivito [36] showed, suggested that the tensile behaviour of more sustainable cementitious composites is influenced by the textile geometry and the volume fraction of fibres. Additional challenges linked to the use of flax fibres are related to their vulnerability to environmental agents such as hygrothermal aging and loading, which affects the prediction of respective lifetimes [32].

In the context of these challenges, impregnations (or coatings) are known to play an important role in the textile mechanical behaviour (e.g., [32,35,37]). For example, Ferrara et al. [37] investigated the influence of impregnation on the morphology of flax fibres, their mechanical properties, and adherence in a hydraulic–lime-based mortar and found that the employed impregnation procedure, although not improving the fibres-to-matrix bond, leads to a standardisation of the yarn’s morphology and reduces the yarn’s deformability in tension. However, despite the relevance of these investigation, they exclude the use of resins with (at least some) bio-based resins. Bio-based resins are known to have less negative impact on the environment instead of the use of conventional polymeric or mineral impregnations [38]. Another drawback identified in the current state of the art concerns the limited knowledge about the use of leno weaves in the context of natural flax fibres. However, leno weaves are already known in the field of mineral fibres (e.g., basalt). For example, Zhang et al. [39] explained that basalt fibres used for producing leno textiles have slightly better mechanical properties than those of AR-glass fibres, present excellent chemical and thermal stabilities and are low-cost and environmentally friendly.

This paper sits in the context of a research project named “Investigation of sustainable reinforcements made of natural fibres for textile concrete components (laboratory phase)” promoted by the German Federal Environmental Foundation (DBU) (in German: Deutsche Bundesstiftung Umwelt) with the aim to investigate the properties and load-bearing behaviour (in terms of tensile and bending loads) of cement-based matrices reinforced with textiles made of bio-based impregnated leno fabrics [40]. This paper focuses on the results of experimental tensile tests on leno fabrics with bio-based impregnations. For comparison, non-impregnated textiles were also tested and investigated. The results presented and discussed address the influence of three parameters—(i) impregnation, (ii) fineness of the weft yarns and (iii) the opening width of the warp yarns (further described in Section 2.1)—on the tensile load behaviour of concrete components reinforced with leno fabrics made of flax fibres. This investigation contributes to the understanding of the tensile load-bearing behaviour of leno fabrics made of flax fibres impregnated with a bio-based resign and, thus, sets avenues to enable the practical application of these textiles as a reinforcement in concrete members.

## 2. Experimental Program

### 2.1. Influencial Parameters on the Tensile Load Behaviour to Investigate through Testing

As discussed in Section 1, impregnation has an important role on the mechanical behaviour of reinforced members. From the field of conventional synthetic fibres, it is known that the impregnation of a textile mesh/grid using, for example, polymer-based coatings, is often used to eliminate the heterogeneous structure of the yarn [41]. The penetration of the coating into the yarn activates more internal filaments, which, in turn, improves the load transfer between the filaments. Ideally, the aim is to activate the entire yarn to achieve a constant stress distribution across its cross-section [41]. This configuration can be seen as analogous to a uniform bond surface between the concrete matrix and the reinforcement. Previous studies (e.g., [42,43]) have demonstrated that added surface coatings contribute to enhanced bond strength and composite tensile strength. Coatings applied to entire sections of textile mesh/grid can influence the stiffness and draping characteristics. Other benefits include additional surface protection during handling and within an alkaline environment; thus, preventing (or at least reducing) the process of fibre decomposition (or degradation) to a minimum, and contributing to the longer durability of a component [38].

Investigating the fineness of weft yarns is important to understand their influence on the mechanical performance of a composite due to variation of the surface–volume relationship [44]—and, ultimately, due to variation of the reinforcement ratio. In this context, yarn fineness is defined as weight per unit length which is also known as linear density in terms of textile technology. Yarn fineness is measured in tex (g/1000 m) [41]. In the field of hemp fibres (also a type of natural fibres) fineness is known to affect the surface area of the fibre and the interfacial bond strength [45]. Furthermore, in conventional synthetic fibres (for example, polymer reinforcements), the strength of a polymer is directly dependent on not only the interfacial bond strength between the fibres and matrix, but also the tensile strength of the fibres themselves [46]. Hence, for the sake of this investigation, it is assumed that a relationship exists between the tensile strength properties of the concrete members and the variation of yarn fineness.

The geometric characteristics of a textile mesh/grid in terms of the opening width of the warp yarns (i.e., spacing of the open structure) is also known to influence the mechanical behaviour of a concrete member. From the field of conventional synthetic fibres, it is also known that the textile mesh/grid spacing primarily influences the cracking process (e.g., crack spacing and width), while the stability of the textile, as well as the strength and stiffness of the cross-points, can influence the bond strength [41]. Note that the opening spacing also influences the quality of concrete pouring.

### 2.2. Methodology

To evaluate the load-bearing capacity of the composite, knowledge of the tensile strength of the reinforcement is required. In this investigation, 39 tests were conducted on test specimens with axial reinforcement made of flax fibre textiles. For comparison, the weft yarns made of flax fibres were characterised through tensile tests performed according to ASTM D 2256/DIN EN ISO 2062 [47]. Due to a premature failure of the impregnated yarns in the clamping areas, the clamping was slightly adjusted based on the recommendations of [48]. Hence, the yarns were glued in steel sleeves with epoxy resin (further characterised in Section 2.3.2) on both sides. In this way, the impregnated yarns were clamped in the testing machine with the steel sleeves and, therefore, premature failure was prevented. For the concrete characterisation, the provisions of DIN EN 196-1 [49] were adopted.

### 2.3. Material Properties

#### 2.3.1. Concrete

For this research project, a tailor-made concrete mixture was developed based on the results of previous research projects involving conventional synthetic fibre textiles embedded in cement-based matrices (e.g., [6,50,51,52,53,54,55]). The goal of the mixture was to meet the requirements of satisfactory workability (i.e., high flowability and penetration of the fabric), as well as to guarantee satisfactory mechanical properties by means of relatively low tensile strength and normal compressive strength. It should be highlighted that the maximum grain size utilised in this investigation (i.e., 2 mm) was due to the limited opening width of the textiles (max. 15 mm in each direction, Section 2.3.2). Thus, a standard concrete mixture, defined according to the provisions of DIN EN 206 [56], could not be used in the context of this investigation. Table 1 characterises the concrete mixture in terms of composition and characteristic mechanical properties.

For this investigation, six prisms with dimensions $l\times w\times h_{b}=160\times 40\times 40$ mm^3^ were cast according to the provisions of DIN EN 196-1 [49] alongside every tensile specimen series. To determine the mean flexural and the mean compressive strength of the concrete mixture, the prisms were stored identically to the tensile specimen and tested at the same age. The uniaxial tensile strength $f_{ctm}$ was calculated using the provisions of *fib* Model Code for Concrete Structures 2010 [57] from the mean value of the flexural tensile strength $f_{ctm,fl}$, which was determined according to the provisions of DIN EN 196-1 (Equation (1)):

(1) $$f_{ctm}=\alpha_{fl}\times f_{ctm,fl}=\frac{0.06\;h_{b}^{0.7}}{1+0.06\;h_{b}^{0.7}}\;f_{ctm,fl}$$

#### 2.3.2. Textiles

**Figure 1:**
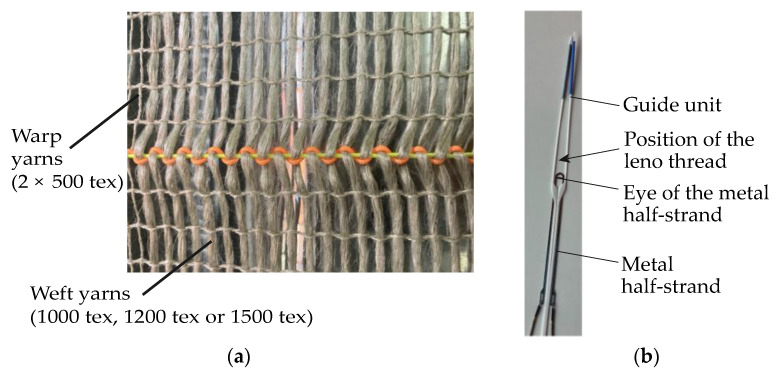
(**a**) Exemplary leno fabric with leno (orange) and standing (neon yellow) yarns in warp direction and weft yarns orthogonal to them; (**b**) Leno heald for producing the twist of the warp yarns in leno fabric.

The textiles used in this investigation consisted of flax fibre yarns produced at the facilities of Fraunhofer Institute for Wood Research (Wilhelm-Klauditz-Institut WKI in Germany). They were produced as a leno fabric with a double-rapier weaving machine with Jacquard attachment. The production process of the textiles is described as follows: In a leno fabric, two yarns are combined in the warp direction. These are (i) the ground yarn that runs at right angles to the weft yarn, and (ii) the leno yarn that turns 180° around the standing yarn after each weft yarn and then encloses it (Figure 1a). This twist is created by special healds—the so-called leno healds—through which the warp material passes. The standing yarn is in the eye of the metal half-strand and the leno yarn sits above it in the guide unit of the entire leno strand. This strand is made of plastic (Figure 1b).

By moving the strand up and down, a lift is created that moves the leno yarn to the right and left, creating the rotation. Due to the twist, leno fabrics can be more stable (i.e., more resistant to displacement) than other conventional 2D fabrics (e.g., plain-weave fabrics) when producing the wide yarn spacings required for this investigation. During the weaving process, the undulation of the weft yar ns is minimised to reduce the yarns’ damage [58].

**Figure 2:**
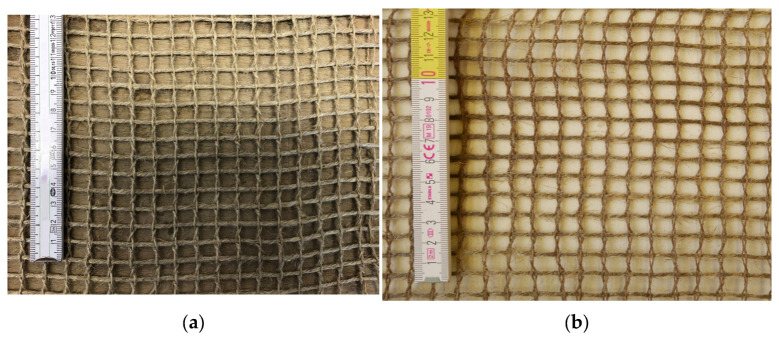
Flax fibre textiles used for the tensile tests (weft yarns horizontally aligned): (**a**) Non-impregnated (NI); (**b**) Impregnated (N).

The textiles used in the test campaign consist of a weft yarn with various levels of fineness: 1000 tex, 1200 tex, and 1500 tex. In the warp direction, the standing and leno yarns have a fineness of 500 tex each. The textiles have opening widths of 8 mm and 15 mm in the weft direction and 10 mm and 15 mm in the warp direction (Figure 2). For comparison purposes, textiles with a bio-based impregnation and without impregnation were tested. For the impregnation, a bio-based epoxy resin was used, comprising 56 % of its molecular structure of renewable origin. The geometry of the textiles, the results of the tensile tests on single yarns (determined according to the provisions of [47]) and the properties of the impregnation are characterised in Table 2.

The different values attained for the elongation of the impregnated textiles should be explained here. As it is listed in Table 2, the elongation values of the 1000 tex yarns somewhat differ from those of the 1200 tex and 1500 tex yarns. The differences between the elongations registered by the different textiles are due to the different clamping mechanisms utilised in the test campaign. For the 1000 tex yarn series, the provisions of ASTM D 2256/DIN EN ISO 2062 were adopted. For the 1200 tex and 1500 tex yarn series, the yarns were glued in steel sleeves with epoxy resin on both sides (see Section 2.4). Thus, these resulting elongation values cannot be directly compared.

An additional clarification is that the 1000 tex yarns were produced out of two yarns with 500 tex each. These textiles showed smaller tensile strengths than one regular 1000 tex yarn. Furthermore, the tensile strength test of the impregnated 1000 tex yarns resulted, in most cases, in a failure around the clamping area. Due to the limited resources available, no additional leno fabrics could be produced; therefore, preventing further investigations on impregnated and non-impregnated textiles with this fineness.

### 2.4. Test Specimens

**Figure 3:**
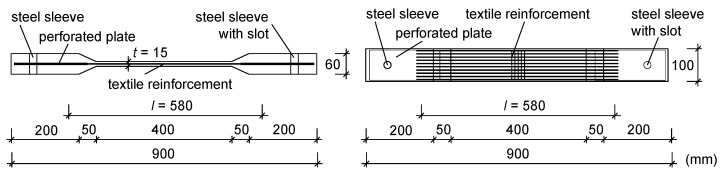
Geometry of the test specimen for the tensile test.

The tensile tests were conducted on bone-shaped test specimens that were already used in comparable tests on concrete components with conventional synthetic fibres [8,59,60,61,62]. Since the non-impregnated textile made of natural flax fibres has a larger cross section than textiles made of conventional synthetic fibres, the web thickness in the free strain length was increased from 10 mm to 15 mm. Hence, the influence of the impregnation and the fineness of the weft yarns could be systematically investigated. The geometry of the test specimens is shown in Figure 3. Note that the external dimensions of the specimens were kept constant during the test campaign, independently of the number of reinforcement layers applied. To consider the unavoidable scatter and obtain reliable mean values, each test configuration was repeated three to four times; thus, a corresponding number of specimens was produced (Table 3). Note that single layers were not investigated since the minimum reinforcement ratio was not reached. This meant that more than one layer was needed to provide a sufficient reinforcement cross-section.

Prior to concreting, the textiles were glued on perforated steel plates (t = 2 mm, Figure 3) to ensure that the reinforcement could be installed centrically and orthogonally to the expected crack direction and, ultimately, avoid anchorage failure. Note that when gluing the non-impregnated textiles, the inner structure of the yarns is influenced. Consequently, higher tensile strengths might be achieved in the concrete compared to that with non-impregnated yarns. Since non-impregnated textiles are less dimensionally stable than impregnated textiles, they were subjected to a manual pre-tension while being installed in the formwork. The main purpose of this procedure was to guarantee that the non-impregnated textiles were aligned (i.e., displayed in a straight manner) during the concrete pouring.

**Figure 4:**
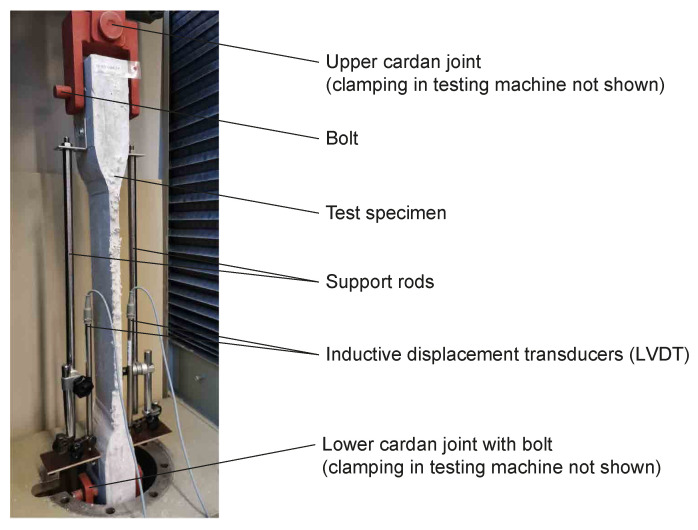
Test setup for the tensile tests with concrete members reinforced with textiles made of natural flax fibres.

All the specimens were removed from the formwork one day after concreting and stored under water until the 14th day to avoid early cracking. Afterwards, they were stored under constant conditions (i.e., 20 °C, 60% relative humidity) until the 28th day. To avoid bending effects in the tensile tests, cardan joints were used. Furthermore, the force and the displacement were continuously recorded using a load cell and two inductive displacement transducers (LVDT) located on the lateral side of the test specimens (Figure 4). Following the provisions of [61], the measurement length was set to 580 mm, which equated the spacing between the brackets on which the LVDTs were installed. To determine the strain, the measurement length was planned to be equivalent to the free strain length; hence, reduced to 400 mm. However, the measurement length was maintained at 580 mm, both to install the LVDTs and to calculate the strains (considering the occurrence of the prior failure of the test specimen and the additional cracks next to the free strain length). Thus, it was ensured that all the occurring strains were considered. This setup also enabled the tracing of the openings of cracks in the wider part and the elastic deformation of the uncracked concrete in the clapped zones. A similar test procedure was adopted by [50,61]. Nevertheless, it is important to highlight that caution is required in the direct comparison of the strains to that in uniaxial tensile tests on pure yarns.

All the tests were conducted in a displacement-controlled manner. Up to 1000 N, the force was applied by 1 mm/min. By applying the pre-load, the starting effects of the testing machine could be easily recognised in the resulting testing diagrams. The speed increased to 5 mm/min. This speed increase was due to the preliminary tests described in [55]. Additionally, the trials with non-impregnated textiles described in [52] showed significantly lower stresses compared to textile reinforcements made of carbon or AR-glass fibres (e.g., [52,62]) at the same elongation level due to the lower modulus of elasticity of the flax fibre yarns. These test specimens had a similar reinforcement amount to those tested in this investigation (e.g., [52]). Other previous studies on the tensile behaviour of textile-reinforced concrete components used even higher test speeds that vary between 10 and 15 mm/min (e.g., [63,64]).

## 3. Test Results

### 3.1. Failure Modes

**Figure 5:**
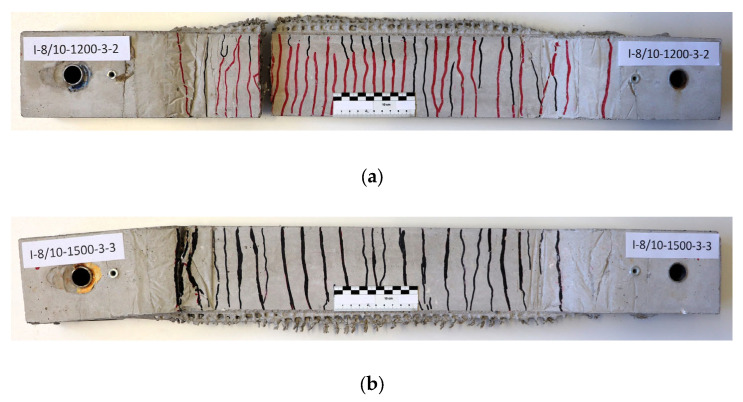
Observed failure modes: (**a**) planned failure with final rupture in the free measuring length; (**b**) unplanned failure with final rupture in the thickened area.

**Figure 6:**
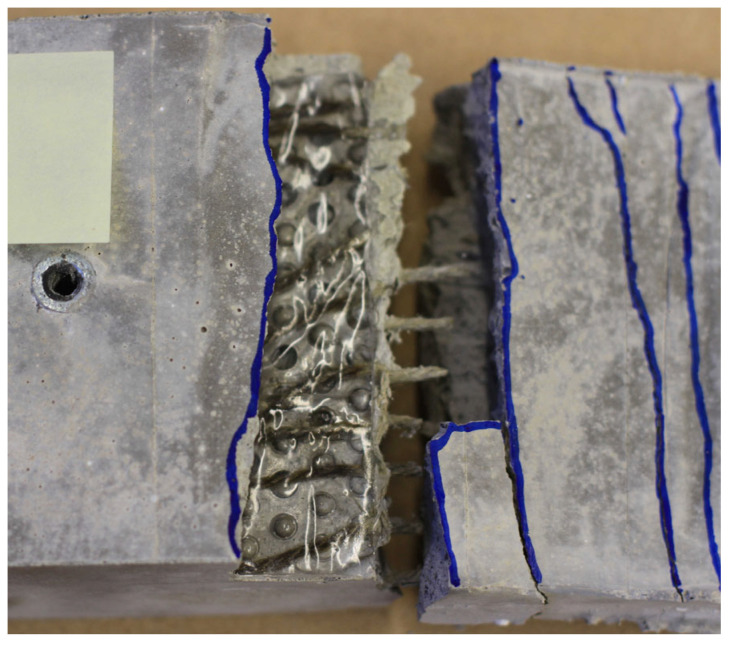
Plain surface due to the epoxy resin and steel plates.

During the tensile test campaign, two main failure mechanisms were observed. First, a rupture of the reinforcement occurred in the free measuring length of the test specimen (i.e., in the thin web) (Figure 5a). Since this failure was expected, it was used as a reference for the analysis of the results. Second, a textile rupture occurred within the transition of the cross-section between the web and the thicker part of the test specimen or in the thicker part itself (Figure 5b). Since this failure was unexpected, it could only be partially used to draw conclusions regarding the tensile load-bearing behaviour. This premature failure was due to the steel plates causing notch stresses and due to the poor bond behaviour between the smooth epoxy-covered zone on the steel plates, the concrete matrix and, ultimately, the textile reinforcement (Figure 6). Such behaviour was not identified during preliminary tests conducted with this reinforcement type [55,62]. It should be stressed here that failure in the free measuring length could occur due to either shortening the steel plates or thickening the heads of the test specimen. Alternatively, if only impregnated textiles are used, there should be enough bond capacity to install them without any steel plate or gluing. To investigate this aspect, further investigations shall be conducted according to the recommendations of Schütze et al. [65].

**Figure 7:**
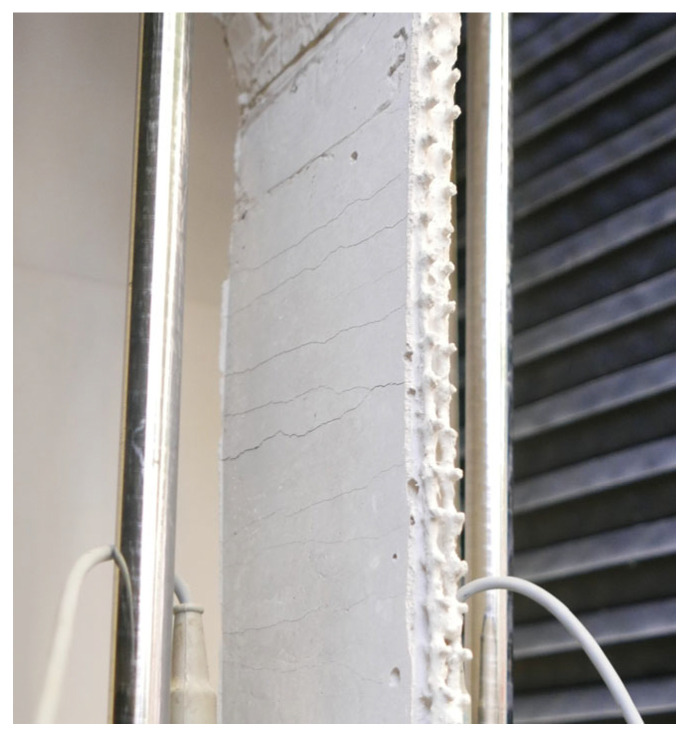
Finely distributed crack pattern with equal crack width of a test specimen during testing.

In both failure modes, the ultimate rupture was brittle, although the failure was pre-announced by finely distributed crack patterns with open cracks, which indicate a certain degree of ductility (Figure 7). The post-failure behaviour was caused by the finite-length yarns tearing or being pulled apart. In light of these results, it can be argued that this represents a successive failure of the individual yarns.

### 3.2. Stress–Strain Behaviour

Table 4 lists the results of the experimental tests, namely the first stress–strain values when the first crack occurred as well as the ultimate state values.

**Figure 8:**
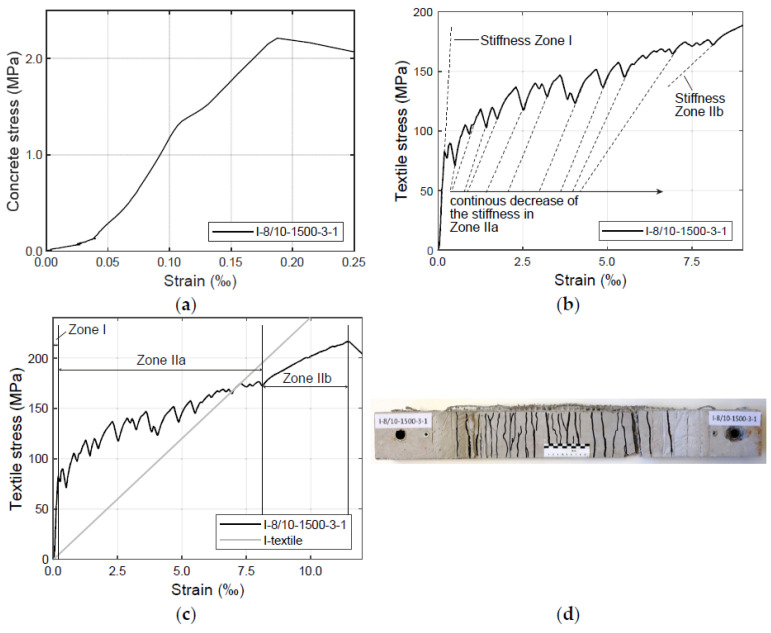
Stress–strain diagrams of the specimen with an impregnated textile (1500 tex fineness of weft yarns): (**a**) Zone I; (**b**) Zone IIa; (**c**) Zones I, IIa and IIb; (**d**) crack pattern of test specimen.

The stress–strain diagrams displayed in Figure 8 were derived from the relationship between the force and deformation of the LVDTs. This relationship was determined according to the provisions prescribed in [65]. The stress of the textiles in the concrete was calculated by dividing the corrected machine force (i.e., considering the starting effects of the testing machine) by the effective cross-section of the textile (described in Table 2). Note here that the reinforcement cross-section was calculated for the pure fibre (i.e., without impregnation). As described in Section 2, due to the external LVDTs, the actual textile strain cannot be directly measured. This strain was determined as the ratio of the mean displacement to the reference-measuring length, which was set to 580 mm. The mean strain of the composite is shown on the axis of abscissas in Figure 8a–c.

The test results displayed in Figure 8a–c refer to one specimen with a three-layer impregnated specimen (1500 tex yarns in the weft direction). The results of the test on this specimen yielded the highest increase of the force and generated a fine crack pattern that should be analysed here. From Figure 8, it is possible to identify a typical tensile stress–strain curve of a reinforced concrete component. The course of this curve is comparable to the findings described in previous investigations such as, for example, the well-known ACK model proposed by Aveston–Cooper–Kelly [66,67]. The ACK model is based on an analytical approach to inorganic-based composites and defines the theoretical stress–strain behaviour of a composite with a brittle matrix in which the fibre–matrix bond remains intact after the matrix has cracked [68]. In Figure 8, as in the ACK model, a non-linear curve is identified which can be divided into three zones: the pre-cracking zone (Zone I) (Figure 8a), the multiple cracking zone (Zone IIa) (Figure 8b) and the post-cracking zone (Zone IIb) (Figure 8c).

In the pre-cracking zone (Zone I) (Figure 8a), the stress is mainly supported by the concrete as there are no cracks; here, the reinforcement has not been activated yet. The stiffness of the uncracked specimen is dependent on the stiffness of the concrete matrix. This zone ends at the point where the first crack appears.

In the multiple cracking zone (Zone IIa) (Figure 8b), the first crack of the concrete cross-section occurs. Similar to the course of the curve in the ACK model [66,67,68], within Zone II, the stresses are supported by the concrete between the cracks and the reinforcement. When the tensile strength of the concrete matrix is exceeded, the first crack is formed and the whole tensile force is carried by the reinforcement in the crack, which is—in this zone—able to resist the acting load. With the increase of the tensile force, new cracks appear in the specimen. Due to the bond between the fibre yarns and the concrete matrix, forces are initiated again in the matrix. When the tensile strength of the concrete is reached once more, a new crack is formed. As can be seen also in Figure 8b, the stiffness of the specimen at the beginning of Zone IIa is approximately as high as in Zone I. With the progressive formation of cracks, the tension-stiffening effect reduces and, consequently, the stiffness of the specimen decreases. At the end of Zone IIa, the stiffness approaches that of Zone IIb [8]. According to [69], the distance between the cracks and their width is influenced by the reinforcement cross-section, the reinforcement ratio, the reinforcement–matrix bond behaviour and the tension failure strain of the concrete matrix. Zone IIa ends when no further cracks occur.

As soon as no further crack occurs (i.e., when a stabilised crack pattern is visible [69]), the post-cracking zone (Zone IIb) is initiated (Figure 8c). When all the cracks are formed, the cracked material behaves in a linear way again (with a lower slope than that obtained in Zone I) and the textile only carries extra load up its maximum tensile stress. Note that the stiffness at this stage is usually lower than the elastic modulus of the textile reinforcement. Hence, the curve in Zone IIb is flatter than the stress–strain curve of the textile under pure tensile load [69]. A possible explanation is the loss of adhesive bond between the impregnated fibre yarns and the concrete matrix [52].

It should be highlighted here that the derivation of an analytical model similar to the ACK model was also established on the basis of the results presented and discussed in this investigation. This model cannot not be discussed here considering that this investigation focuses on the analysis of the three above-mentioned influential parameters alongside the inherent length constraints of this manuscript. However, this newly derived analytical model is available and can be consulted in [70].

A last aspect to highlight from the set of results listed in Table 4 is that, at the ultimate state, the specimen reinforced with a textile made of natural fibres registers similar or even lower elongations compared to carbon or AR-glass textile specimens with an equal reinforcement ratio; however, at a significantly lower stress-level. In [52] the tensile, the strength of specimens reinforced with impregnated carbon fibre textiles was examined. For the specimen V3-T11-S2 (reinforcement ratio: 2.35%, fineness of the weft yarns: 1650 tex), a mean ultimate stress of 3156 MPa with a mean elongation of 12.6‰ was measured. By analysing the specimens in this study with a similar reinforcement ratio, it can be observed that the test series I-8/10-1200-3-x (reinforcement ratio: 2.14%) registered a mean ultimate stress of 223 MPa at a mean elongation of 10.8‰. Furthermore, specimens with impregnated AR-glass textiles were also tested in [52] (e.g., test series V3-T91-K5 with reinforcement ratio: 1.29%, fineness of the weft yarns: 2400 tex). These tests registered a mean ultimate stress of 1088 MPa and a mean elongation of 15.8‰. The corresponding test series of this study, I-8/10-1000-2-x (reinforcement ratio: 1.19%), resulted in a mean ultimate stress of 219 MPa with a mean elongation of 6.9‰.

## 4. Discussion

### 4.1. Influence of the Impregnation

**Figure 9:**
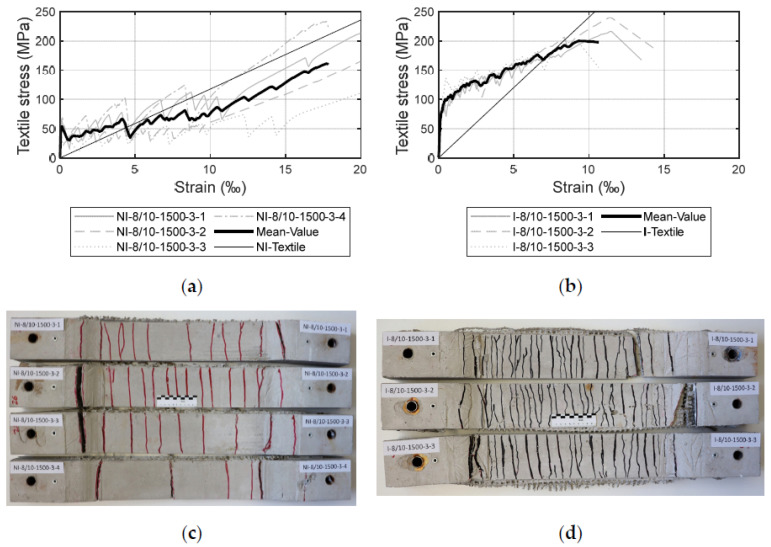
Stress–strain diagrams and crack patterns of specimen with 1500 tex fineness of weft yarns and three layers of reinforcement: (**a**,**c**) non-impregnated textiles; (**b**,**d**) impregnated textiles.

Figure 9 compares the results of the failure stresses and the crack pattern of specimens with a three-layer reinforcement without impregnation (Figure 9a,c) and with impregnation (Figure 9b,d).

By comparing the stress–strain curves of the impregnated and non-impregnated textiles, the mean values of the strains are lower with impregnated textiles. This behaviour is also visible on the tensile curves of the yarns. The larger strains with non-impregnated textiles can be explained by the fact that the fibres needed to be pre-tensioned to be activated; thus, there is only a sliding friction between the fibres in the yarn. In comparison, the impregnation homogenises the cross-section of the yarn and ensures that the fibres are bonded together, leading to reduced strain due to earlier activation, and enabling higher stress levels. Other possible reasons for the lower strains and the higher stresses could be the improved alignment of the reinforcement (i.e., the textiles are dimensionally more stable) before and during the concreting process.

Also, the stiffened intersection between the impregnated weft and warp yarns has primarily a positive effect on the bond behaviour in the fibre–matrix interface. Regarding the stiffness of the specimen in Zone IIb, the specimen with the non-impregnated textile has approximately equal stiffness to that of the pure yarn. Since impregnated textiles have a smoother surface than the non-impregnated ones, it facilitates the transition from adhesive to sliding bond in Zone IIb and, thus, to a lower stiffness than the pure impregnated yarn [52]. Impregnated textiles were able to transfer load more easily, as their weft yarns were aligned more orthogonal to the directions of the cracks. Since the impregnated textiles are dimensionally more stable, they are activated at a lower strain state. Also, the impregnation provided a satisfactory bond between the fibres and the matrix interface. The resin was able to penetrate the inner fibres and prevented slippage between the inner and outer fibres.

The specimens reinforced with impregnated textiles demonstrated a more finely distributed crack pattern compared to those reinforced with non-impregnated textiles. When using impregnated textiles, they were finely distributed over the whole test specimen, which opened similarly during the testing. Overall, it can be argued that these results support the assumption that the inner frictional interface stress increases with the impregnation, meaning that practically every fibre contributes to the load bearing and, thus, the impregnated textiles can be better activated. The impregnation also helps to nullify the influence of small defects in the yarns caused, for example, by the weaving or reaping process. Ultimately, the load-bearing capacity of the whole component increases.

The above-described phenomena are not only valid for the specimen analysed in Figure 9. The results in Table 4 show that the impregnated textiles enable a slightly higher range of failure stresses as well as much smaller strains in comparison to those yielded by the textiles without impregnation. For example, for the test series NI-8/10-1200-3-x, the mean value for the failure stress is 217 MPa, whereas for the corresponding impregnated specimens (i.e., I-8/10-1200-3-x), the failure stress registered a mean value of 223 MPa. The difference for the strains is more pronounced at the ultimate state: 16.4‰ and 10.5‰, respectively, for non-impregnated and impregnated textiles.

### 4.2. Influence of the Fineness of the Weft Yarns and the Reinforcement Ratio

**Figure 10:**
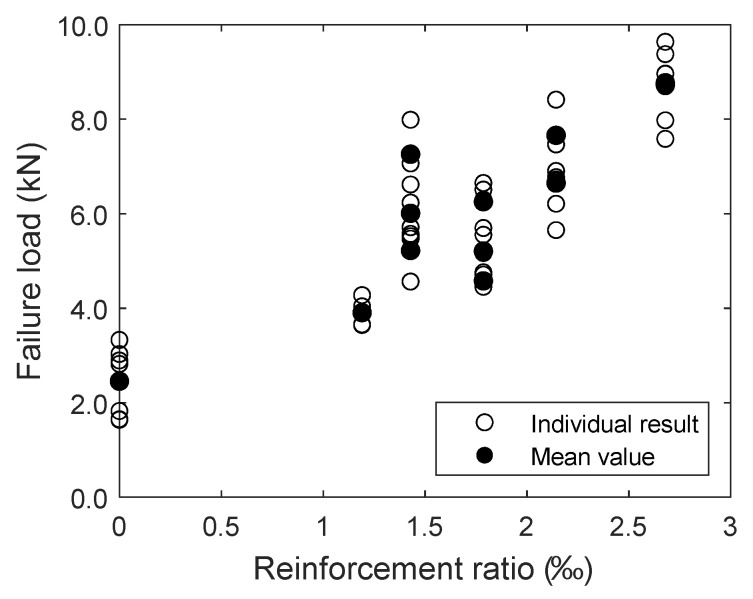
Failure loads of a test specimen in dependence of the reinforcement ratio.

In this investigation, the influence of the fineness of the impregnated weft yarns was analysed for 1000 tex, 1200 tex and 1500 tex yarns. Note that, for the sake of this investigation, an increase in the fineness means an increase in the reinforcement cross-section. The results listed in Table 4 and displayed in Figure 10 show that an increase in the reinforcement ratio leads to an increase in the failure load. From Figure 10, it is also visible that the failure load only increases significantly—in comparison to a specimen without reinforcement—if a minimum reinforcement amount is provided.

**Figure 11:**
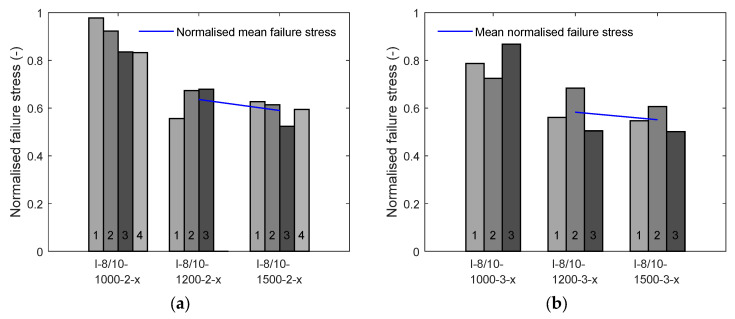
Normalised failure stresses of the test specimens I-8/10-1000, I-8/10-1200 and I-8/10-1500 (numbers 1 to 4 refer to the test number within the respective test series): (**a**) two layers of impregnated textiles with varying fineness of weft yarns; (**b**) three layers of impregnated textiles with varying fineness of weft yarns.

Figure 11 represents the ultimate normalised stresses plotted against the fineness of the textiles. To eliminate the different tensile strengths of the yarns, the ultimate stresses observed in the tests were divided by the tensile stress of the impregnated yarns. As mentioned in Section 2, the tensile strength of the impregnated yarns with 1000 tex was determined with the non-adjusted test setup. This has led to extremely low values for the tensile strength due to the influence of the clamping on the results. As it can be observed in Figure 11, the specimens with 1000 tex weft yarns have much higher nominal failure stresses than those registered from the other investigated levels of fineness, which can be explained by the above-mentioned influence of the test setup. Consequently, these specimens were excluded for the investigation of the influence of the fineness.

When comparing the specimens with 1200 tex and 1500 tex weft yarns, the ultimate normalised failure stresses are within the same range. This applies to the specimens with two- and three-layer reinforcement. The normalised mean failure stress seems to slightly decrease with an increase in fineness. As the fineness increases, the ratio of outer surface area to the cross-section decreases. The test specimens with a smaller fineness have a larger bond surface and, thus, can be better activated. According to Figure 11, the normalised mean failure stress sits between 60% and 65% (approximately) for the investigated two-layer reinforcement and between 55% and 60% (approximately) for the three-layer counterpart. These results seem to be in line with the findings of previous investigations in the field of conventional synthetic fibres. For example, for impregnated textiles, Kulas [48] found that the normalised mean failure stress sits around 84%. The slightly higher value can be explained by the shape of the yarns investigated by Kulas, which have a larger outer surface area. For non-impregnated reinforcement, Voss [57] registered normalised mean failure stresses between 22% and 33%, whereas Molter [46] registered higher activation between 50% and 80%.

Despite these results, it can be argued that the influence of the fineness on the tensile load-bearing capacity appears to be minor. However, to derive more robust conclusions regarding the influence of the fineness, further tests shall be conducted.

### 4.3. Influence of the Opening Width of the Warp Yarns

**Figure 12:**
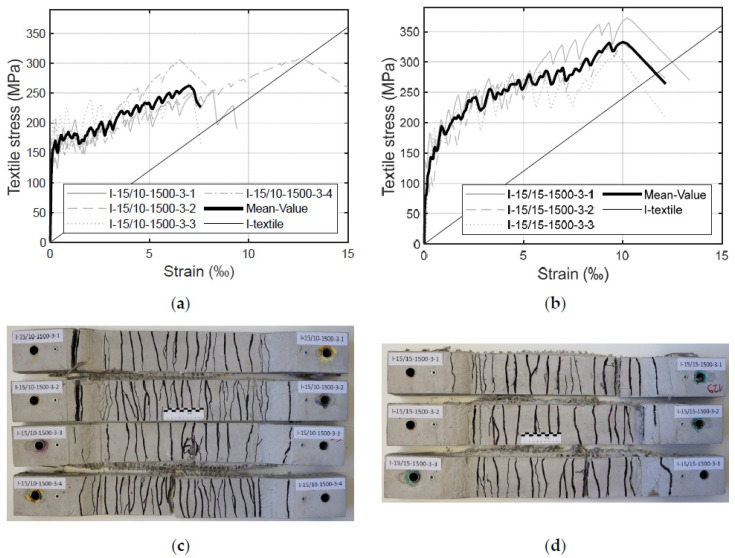
Stress–strain diagrams of test specimen with impregnated textiles for: (**a**) 10 mm opening width of the warp threads; and (**b**) 15 mm opening width of the warp threads; (**c**) crack pattern of test specimens with textiles with 10 mm opening width of the warp threads; (**d**) crack pattern of test specimens with textiles with 15 mm opening width of the warp threads.

The influence of the opening width of the warp yarns on the load-bearing behaviour of the specimens was also investigated. For this purpose, two test series were conducted with similar impregnated textiles to those described above in Section 4.1 and Section 4.2. The test series only differed in terms of the spacing between the warp yarns: 10 mm and 15 mm. The stress–strain curves and the corresponding crack patterns are visible in Figure 12.

Figure 12 shows that, with a spacing of 15 mm, a higher failure stress level can be attained. The test specimens with an opening width of 10 mm (Figure 12a) failed between 250 MPa to 310 MPa, whereas the test specimens with a warp yarn spacing of 15 mm (Figure 12b) reached an ultimate stress level in the range between 310 MPa to 370 MPa. These results indicate a better utilisation of the textile reinforcement with 15 mm spacing between warp yarns; also, the weft yarns seem to be better activated. This behaviour might be explained by the fact that the textiles are less damaged during the production since there are less warp yarns interfering with the weft yarns. The more warp yarns the textile has, the more undulation the weft yarns have, and the worse the yarn alignment is.

Larger spacing between the warp yarns increases the spacing between the cracks, which leads to fewer cracks than in textiles with smaller spacing (compare Figure 12c,d). Nevertheless, the crack pattern remains finely distributed (Figure 12d). This suggests that the warp yarns may be promoting cracking, which is also a known phenomenon from conventional synthetic fibre fabrics (e.g., glass or carbon fibres) (e.g., [8]). The yarns cannot support any further load as they are aligned parallel to the cracks and, therefore, even benefit the cracking process.

The stiffness of concrete members in Zone IIb is comparable to the stiffness of the pure textiles. Considering the results described in Section 3.2, this statement is valid for both opening widths adopted in this investigation: 10 mm and 15 mm. However, the effect can be more clearly observed for wider spacing between the warp yarns. In addition, the strains of the specimens with an opening width of 15 mm are lower than the strains of the corresponding yarns. Here, no “stiffness/deficit” can be observed, which emphasises the considerations described in Section 3.2, where the combination of various effects might influence this behaviour. In terms of various opening widths between the warp yarns, a wider spacing seems to reduce the “stiffness/deficit” and improve the uniaxial load-bearing behaviour of the component compared to the pure textiles and the test specimens with narrower spacings. An explanation for this phenomenon could be that, for larger opening widths, the transition from adhesive bond to friction bond takes place at higher stresses.

Furthermore, the effects of tension stiffening in Zone IIa and Zone IIb can also be observed. Here, the strains of the components are lower than the strains of the single yarns. This suggests that the concrete does not allow for the free elongation of the textile reinforcement between the cracks, as described in [8]. This effect is more distinct for spacings of 15 mm between the warp yarns. A possible explanation could be that the warp yarns in smaller spaces might weaken the concrete cross-sections and lower the resistance to cracking; thus, more cracks appear. In comparison, textiles with larger spacing have fewer cracks; therefore, the distance between adjacent cracks increases. Since more tension can be transferred from the reinforcement to the concrete, the transfer length between two cracks also increases. It is important to highlight here that larger spacing enables the use of a larger grain in the concrete mixture, which is common practice in the building industry. Also, with larger opening widths, there is less material consumption as the warp yarns do not bear the load when the textiles are used only for uniaxial loads. However, it is important to exercise caution when recommending large grain sizes in a concrete mixture since this would have implications on the concrete cover (i.e., possibly a larger concrete cover would be required to guarantee a satisfactory bond strength). Additional challenges related to the penetration of the multilayer reinforcement could occur. The above-described advantages can be unlocked only for impregnated textiles since, as stated above, the non-impregnated textiles are less stable. As it was previously explained, non-impregnated textiles with similar opening widths cannot be installed as easily as impregnated textiles. For dimensionally more stable impregnated textiles, the reduced interfering effects of fewer warp yarns enable a better performance of the reinforcement.

## 5. Conclusions

### 5.1. Current State of Advancement

The present investigation led to the following conclusions:

- Overall, the results of the tensile tests presented in this paper suggest that textiles made of flax fibres (as a leno fabric) impregnated with a bio-based resin have the potential to be used as a reinforcement for uniaxial tension in concrete-based members;
- The results of this investigation showed that specimens with reinforcements made of flax fibres are capable of withstanding higher failure loads than those supported by specimens without reinforcement. Yet, a minimum reinforcement amount is necessary;
- Additionally, the results indicate that the failure mode is mostly brittle due to the linear elastic behaviour of the textiles. However, the failure is clearly announced by a finely distributed crack pattern and large strains;
- Regarding the stress–strain behaviour of the test specimens, three zones were identified: pre-cracking zone (Zone I), multiple cracking zone (Zone IIa) and post-cracking zone (Zone IIb). These zones are typical for textile-reinforced concrete members under tension load;
- The impregnation seems to enable a more finely distributed crack pattern than that on the specimens without impregnation. The level of bond at the fibre–matrix interface was better in the impregnated textiles since the resin was able to penetrate the inner fibres and avoid slippage between the inner and the outer fibres;
- Additionally, high failure stresses were registered for the impregnated textiles in comparison to the specimens without impregnation. Impregnation leads to dimensionally more stable reinforcement and, consequently, to better activation. The impregnation also minimises the influence of small defects in the yarns implemented during the weaving or reaping processes. Overall, the tensile load-bearing capacity is improved with impregnation;
- No clear influence of the fineness of the weft yarns on the tensile load-bearing capacity could be determined. It appears that the load-bearing capacity decreases slightly with greater fineness. However, for more robust conclusions, further tests should be conducted;
- The distance of the warp yarns seems to influence the crack patterns. Smaller opening widths of the warp yarns lead to a finer distributed crack pattern since the warp yarns weaken the specimen cross-section. In comparison, large spacings seem to promote a better activation of the yarns due to the larger transmission length. In addition, higher failure stresses where observed. This could be explained by the reduced ondulation in the textiles since there are fewer warp yarns interfering with the weft yarns.

### 5.2. Future Developments

Considering the highly variable nature of fibre properties, further tests should be carried out on a larger number of textile samples, where distinct properties can be investigated with the goal to verify the statistical significance of the trends observed in this study. A yet unanswered question regards the long-term effect of the natural flax fibres in the alkaline concrete environment on durability, including the influence of temperature (e.g., [71,72]) or moisture content, and other long-term behaviour parameters. Such investigations are needed to understand the extent to which impregnation protects the flax fibre yarns and whether the protection works after exposure to physical effects (e.g., tensile strain, cracks). Additionally, further research work is needed to investigate the behaviour under shear forces and under uniaxial bending. Another relevant work direction for future investigation is in the field of lifecycle cost analyses for practical structural applications, including the comparison with conventional synthetic or mineral fibres.

## Figures and Tables

**Table 1:** Characterisation of the concrete mixture.

Concrete Mixture Composition	kg/m^3^
CEM II 32.5 N/LH	424.7
Sand 0/2 (aggregate size varying between 0 and 2 mm)	1039.1
Limestone powder	707.8
Silica powder	54.4
Superplasticizer	3.6
Water	288.9
**Characteristic mechanical properties of hardened concrete** **(experimental mean values of all test series measured at 28 days)**	**MPa**
Flexural strength (tests according to [49])	6.86
Uniaxial tensile strength (calculated according to [57])	3.03
Compressive strength of concrete (tests according to [49])	47.24

**Table 2:** Characterisation of the flax fibre textiles (gauze weave) and the impregnation.

Spacing and Fineness Properties of Flax Fibre Textiles	Properties					
Spacing of weft yarns (mm) ^(a)^	8; 15					
Spacing of warp yarns (mm) ^(a)^	10; 15					
Fineness of weft yarns (tex)	1000; 1200; 1500					
Fineness of warp yarns (tex)	2 × 500					
Reinforcement cross-section (mm^2^/m) ^(b)^	71.4; 89.3; 107.1; 133.9					
**Impregnation properties**	**Properties**					
Impregnation	SICOMIN Green—Poxy 56					
E-Module	3300 MPa					
Glass transition temperature of impregnation	78 °C					
**Mechanical properties of flax fibre textiles:** **Non-impregnated** **(experimental mean values of 10 tensile tests)**	Tensile strength			Max. elongation		
	Value	SD	CoV	Value	SD	CoV
	(MPa)	(MPa)	(%)	(%)	(%)	(%)
1000 tex yarn ^(d)^	233.8	37.1	15.9	2.80	0.22	7.86
1200 tex yarn ^(d)^	312.8	34.2	10.9	3.30	0.15	4.55
1500 tex yarn ^(d)^	362.6	34.3	9.5	3.90	0.23	5.90
**Mechanical properties of flax fibre textiles:** **Impregnated** **(experimental mean values of 10 tensile tests)**	Tensile strength			Max. elongation		
	Value	SD	CoV	Value	SD	CoV
	(MPa)	(MPa)	(%)	(%)	(%)	(%)
1000 tex yarn ^(d)^	245.1 ^(c)^	31.9	13.0	2.00 ^(c)^	0.28	14.0
1200 tex yarn ^(d)^	382.9	26.0	6.8	1.47	0.18	12.3
1500 tex yarn ^(d,e)^	395.8	14.6	3.7	1.67	0.04	2.4

SD: Standard deviation; CoV: Coefficient of variation; ^(a)^ Measured axis-to-axis. ^(b)^ Values measured on pure fibre content (without resin). ^(c)^ Partially premature failure at clamping. ^(d)^ For the tests on 1000 tex yarn (for both impregnated and non-impregnated), the provisions of ASTM D 2256/DIN EN ISO 2062 [47] were adopted. Only the impregnated tests on 1200 tex yarns and 1500 tex yarns were measured after the clamping adaption described in Section 2.2. ^(e)^ The values for the impregnated 1500 tex yarns were determined from only 9 tensile tests.

**Table 3:** Characterisation of the experimental program.

Test ID	Impregnation	Spacing Weft Yarns (mm)	Spacing Warp Yarns (mm)	Fineness Weft Yarn (mm)	Number of Layers	Reinf. Cross-Sec. ^(a)^ (mm^2^)	Reinf. Ratio ^(b)^ (%)	Number of Tests
NI-8/10-1000-3-x	None	8	10	1000	3	26.8	1.79	4
I-8/10-1000-3-x	GP56 ^(c)^	8	10	1000	3	26.8	1.79	3
NI-8/10-1200-3-x	None	8	10	1200	3	32.1	2.14	4
I-8/10-1200-3-x	GP56 ^(c)^	8	10	1200	3	32.1	2.14	3
NI-8/10-1500-3-x	None	8	10	1500	3	40.2	2.68	4
I-8/10-1500-3-x	GP56 ^(c)^	8	10	1500	3	40.2	2.68	3
I-8/10-1000-2-x	GP56 ^(c)^	8	10	1000	2	17.9	1.19	4
I-8/10-1200-2-x	GP56 ^(c)^	8	10	1200	2	21.4	1.43	3
I-8/10-1500-2-x	GP56 ^(c)^	8	10	1500	2	26.8	1.79	4
I-15/10-1500-3-x	GP56 ^(c)^	15	10	1500	3	21.4	1.43	4
I-15/15-1500-3-x	GP56 ^(c)^	15	15	1500	3	21.4	1.43	3

^(a)^ The cross-section of the reinforcement was calculated for the pure fibre (i.e., without impregnation). ^(b)^ Measured on the weakened part of the concrete cross-section (i.e., 15 mm × 100 mm). ^(c)^ Impregnation with a bio-based epoxy resin (56% of its molecular structure of renewable origin) according to the properties described in Table 2.

**Table 4:** Results of the experimental campaign: first crack and ultimate state.

	First Crack					Ultimate State			
Test Specimen	Force		Stress Related to the Concrete (c) Cross-Section		Total Strain	Maximum Force	Stress Related to the Textile (t) Cross-Section		Total Strain at Maximum Force
	F		A_c_	σ_c_	ε	F	A_t_	σ_t_	ε
	[N]		[mm^2^]	[MPa]	[‰]	[N]	[mm^2^]	[MPa]	[‰]
NI-8/10-1000-3-x	1	2312	1500	1.54	0.12	4569	26.79	170.6	11.09
NI-8/10-1000-3-x	2	3024		2.02	0.13	4454		166.3	18.95
NI-8/10-1000-3-x	3	1941		1.29	0.09	4584		171.1	18.26
NI-8/10-1000-3-x	4	3042		2.03	0.33	4711		175.9	4.03 ^(a)^
I-8/10-1000-3-x	1	1664	1500	1.11	N/A	5167	26.79	192.9	9.65
I-8/10-1000-3-x	2	2392		1.59	0.18	4760		177.7	3.55 ^(a)^
I-8/10-1000-3-x	3	2721		1.81	0.15	5698		212.7	9.18
NI-8/10-1200-3-x	1	3579	1500	2.39	0.18	6772	32.14	210.7	13.72
NI-8/10-1200-3-x	2	3592		2.39	0.18	7467		232.3	19.09
NI-8/10-1200-3-x	3	2337		1.56	0.21	6705		208.6	16.29
NI-8/10-1200-3-x	4	2888		1.93	0.07	5655		175.9	16.58
I-8/10-1200-3-x	1	2392	1500	1.59	0.24	6905	32.14	214.8	11.46
I-8/10-1200-3-x	2	1944		1.30	0.12	8414		261.8	13.02
I-8/10-1200-3-x	3	2341		1.56	0.08	6212		193.3	8.03
NI-8/10-1500-3-x	1	2664	1500	1.78	0.26	8964	40.18	223.1	22.28
NI-8/10-1500-3-x	2	2769		1.85	0.20	8962		223.0	25.27
NI-8/10-1500-3-x	3	2309		1.54	0.13	7586		188.8	28.44
NI-8/10-1500-3-x	4	1706		1.14	0.14	9377		233.4	30.48
I-8/10-1500-3-x	1	3316	1500	2.21	0.21	8699	40.18	216.5	11.42
I-8/10-1500-3-x	2	2471		1.65	0.15	9639		239.9	11.33
I-8/10-1500-3-x	3	3446		2.30	0.17	7977		198.5	9.00
I-8/10-1000-2-x	1	2536	1500	1.69	0.08	4278	17.86	239.5	11.48
I-8/10-1000-2-x	2	2219		1.48	0.12	4042		226.3	7.57
I-8/10-1000-2-x	3	2142		1.43	0.16	3658		204.8	4.73
I-8/10-1000-2-x	4	1981		1.32	0.10	3646		204.1	3.74
I-8/10-1200-2-x	1	2136	1500	1.42	0.04	4565	21.43	213.0	6.41
I-8/10-1200-2-x	2	3443		2.30	0.61	5525		257.8	8.08
I-8/10-1200-2-x	3	2327		1.55	0.13	5573		260.1	10.49
I-8/10-1500-2-x	1	1742	1500	1.16	0.09	6650	26.79	248.2	13.85
I-8/10-1500-2-x	2	1507		1.00	0.08	6510		243.0	12.08
I-8/10-1500-2-x	3	2189		1.46	0.11	5555		207.4	12.51
I-8/10-1500-2-x	4	2936		1.96	0.15	6299		235.1	12.32
I-15/10-1500-3-x	1	3167	1500	2.11	0.15	5472	21.43	255.3	8.18
I-15/10-1500-3-x	2	2691		1.79	0.09	6619		308.9	12.66
I-15/10-1500-3-x	3	4395		2.93	0.20	5715		266.7	7.02
I-15/10-1500-3-x	4	3539		2.36	0.17	6235		290.9	11.25
I-15/15-1500-3-x	1	2962	1500	1.97	0.07	7988	21.43	372.7	10.19
I-15/15-1500-3-x	2	1908		1.27	0.12	7065		329.7	10.03
I-15/15-1500-3-x	3	2509		1.67	0.22	6725		313.8	9.66

NI: Specimens with non-impregnated textiles; I: Specimens with impregnated textiles; 2-x: Test series with two layers of reinforcement; 3-x: Test series with three layers of reinforcement; ^(a)^ Maximum force was reached during Zone IIa (multiple cracking zone).

## Data Availability

Data are contained within the article.
